# Potentiation of Mitogenic Activity of Platelet-Derived Growth Factor by Physiological Concentrations of Insulin via the MAP Kinase Cascade in Rat A10 Vascular Smooth Muscle Cells

**DOI:** 10.1080/15604280214489

**Published:** 2002

**Authors:** Hitomi Yamada, Toshio Tsushima, Hitomi Murakami, Yasuko Uchigata, Yasuhiko Iwamoto

**Affiliations:** 1 Diabetes Center Tokyo Women's Medical University. Kawadacho 8-1 Shinjukuku, Tokyo 162-8666 Japan; 2 Department of Medicine Tokyo Women's Medical University. Kawadacho 8-1 Shinjukuku, Tokyo 162-8666 Japan

## Abstract

Hyperinsulinemia has been shown to be associated with diabetic angiopathy. Migration and proliferation of vascular smooth muscle cells (VSMC) are the processes required for the development of atherosclerosis. In this study, we attempted to determine whether insulin affects mitogenic signaling induced by plateletderived growth factor (PDGF) in a rat VSMC cell line (A10 cells). PDGF stimulated DNA synthesis which was totally dependent on Ras, because transfection of dominant negative Ras resulted in complete loss of PDGF-stimulated DNA synthesis. Initiation of DNA synthesis was preceded by activation of Raf-1, MEK and MAP kinases (Erk 1 and Erk2). Treatment of the cells with PD98059, an inhibitor of MAPK kinase (MEK) attenuated but did not abolish PDGF-stimulated DNA synthesis, suggesting that MAPK is required but not essential for DNA synthesis. PDGF also stimulated phosphorylation of protein kinase B (Akt/PKB) and p70 S6Kinase (p70S6K) in a wortmannin-sensitive manner. Rapamycin, an inhibitor of p70S6K, markedly suppressed DNA synthesis. Low concentrations of insulin (1-10 nmol/l) alone showed little mitogenic activity and no significant effect on MAPK activity. However, the presence of insulin enhanced both DNA synthesis and MAPK activation by PDGF. The enhancing effect of insulin was not seen in cells treated with PD98059. Insulin was without effect on PDGF-stimulated activations of protein kinase B (Akt/PKB) and p70S6K. We conclude that insulin, at pathophysiologically relevant concentrations, potentiates the PDGFstimulated DNA synthesis, at least in part, by potentiating activation of the MAPK cascade. These results are consistent with the notion that hyperinsulinemia is a risk factor for the development of atherosclerosis.


*Int. Jnl. Experimental Diab. Res.*, 3:131-144, 2002
© 2002 Taylor and Francis
1560-4284/02 $12.00 + .00

### Potentiation of Mitogenic Activity of Platelet-Derived Growth Factor by Physiological Concentrations of Insulin via the MAP Kinase Cascade in Rat A10 Vascular Smooth Muscle Cells

HITOMI YAMADA[1], TOSHIO TSUSHIMA[2]*, HITOMI MURAKAMI[2]
YASUKO UCHIGATA[1], YASUHIKO IWAMOTO[1]

Diabetes Center[1] and Department of Medicine 2[2], Tokyo Women's Medical University. Kawadacho 8-1, Shinjukuku, Tokyo 162-8666

*Received: March 7, 2001; In final form: November 20, 2001*

Hyperinsulinemia has been shown to be associated with diabetic angiopathy. Migration and proliferation of vascular smooth muscle cells (VSMC) are the processes required for the development of atherosclerosis. In this study, we attempted to determine whether insulin affects mitogenic signaling induced by platelet-derived growth factor (PDGF) in a rat VSMC cell line (A10 cells). PDGF stimulated DNA synthesis which was totally dependent on Ras, because transfection of dominant negative Ras resulted in complete loss of PDGF-stimulated DNA synthesis. Initiation of DNA synthesis was preceded by activation of Raf-1, MEK and MAP kinases (Erk 1 and Erk2). Treatment of the cells with PD98059, an inhibitor of MAPK kinase (MEK) attenuated but did not abolish PDGF-stimulated DNA synthesis, suggesting that MAPK is required but not essential for DNA synthesis. PDGF also stimulated phosphorylation of protein kinase B (Akt/PKB) and p70 S6Kinase (p70S6K) in a wortmannin-sensitive manner. Rapamycin, an inhibitor of p70S6K, markedly suppressed DNA synthesis. Low concentrations of insulin (1-10 nmol/l) alone showed little mitogenic activity and no significant effect on MAPK activity. However, the presence of insulin enhanced both DNA synthesis and MAPK activation by PDGF. The enhancing effect of insulin was not seen in cells treated with PD98059. Insulin was without effect on PDGF-stimulated activations of pro-

*Corresponding author: Department of Medicine 2, Tokyo Women's Medical University, Kawadacho 8-1, Shinjukuku, Tokyo 162-8666
Fax:+81-3-5269-7326; e-mail: tsushimat@endm.twmu.ac.jp

**tein kinase B (Akt/PKB) and p70S6K. We conclude that insulin, at pathophysiologically relevant concentrations, potentiates the PDGF-stimulated DNA synthesis, at least in part, by potentiating activation of the MAPK cascade. These results are consistent with the notion that hyperinsulinemia is a risk factor for the development of atherosclerosis.**

*Key words*: PDGF, insulin, MAPK, VSMC, atherosclerosis

### INTRODUCTION

Proliferation of vascular smooth muscle cells (VSMC) is one factor responsible for the development of atherosclerosis. A number of growth factors, including platelet-derived growth factor (PDGF), epidermal growth factor (EGF), fibroblast growth factor(FGF) and insulin-like growth factors (IGFs), have been shown to stimulate proliferation of VSMC in vivo or in vitro [1-5]. PDGF appears to play a key role in the development of diabetic angiopathy, because it promotes not only proliferation, but also chemotaxis and proteoglycan synthesis in VMSC [6-8]. Earlier studies have also shown endogenous hyperinsulinemia or prolonged insulin administration to result in development of atherosclerosis-like lesions (reviewed in [9, 10]), although insulin is a weak growth-mitogen. It is possible that insulin is permissive in terms of mitogenic activity of other growth factors. Goalstone et al. recently reported that insulin potentiates PDGF-stimulated DNA synthesis in rat and porcine VSMC [11] by stimulating the prenylation of p21 Ras, suggesting that Ras-mediated mechanisms are involved in the potentiating effect of insulin. However, the mechanism by which insulin synergizes with PDGF is not fullly understood.

Both insulin and PDGF transmit signals through binding to cognate receptors with intrinsic tyrosine kinase activity, and they utilize a number of common signaling pathways [12,13] including the mitogen-activated protein kinase (MAPK) cascade and phosphatidylinositol 3-kinase (PI3K). Activation of the classical MAPK pathway is initiated by ligand-induced activation of the receptor followed by sequential phosphorylation and activation of Ras, Raf, MAPK kinase (MAPKK or MEK) and MAPK (also known as Extracellular signal regulated kinase, Erk). Two isoforms of MAPK, the p44 MAPK (Erk-1) and the p42 MAPK (Erk-2) have been identified in most cells, and it is well established that MAPK plays an important role in regulating cell proliferation or differentiation depending on cell types [14, 15].

PI3K is a heterodimer consisting of an 85-kDa regulatory subunit and a 110kDa catalytic subunit. The 85 regulatory subunit functions as an adaptor which links PI3K to tyrosine phosphorylated proteins such as autophosphorylated PDGF receptors and tyrosine-phosphorylated insulin receptor substrate (IRS), and this association stimulates the kinase activity of the p110 subunit [16]. PI3K-mediated activation of the downstream kinases such as protein kinase B (PKB or Akt) and p70S6 kinase (p70S6K), has been implicated in various cellular responses including proliferation, differentiation, and apoptosis [17, 18].

The goal of this study was to determine whether insulin influences PDGF-stimulated activation of the MAPK cascade and PI3K-mediated signaling in rat VSMC (A10). We demonstrate that physiological concentrations of insulin, a weak mitogen on its own, significantly enhances both DNA synthesis and activation of the MAPK cascade simulated by PDGF without little effect on PI3K-mediated signaling. The effect of insulin was abrogated by blockade of the MAPK cascade, which is consistent with the view that insulin effects are mediated via enhancement of PDGF-stimulated MAPK pathway.

### MATERIALS AND METHODS

### *Materials*

Recombinant human PDGF (PDGF-BB) was purchased from Upstate Biotechnology (Lake Placid, NY). Porcine insulin was obtained from Sigma Chemical (St. Louis, MO), as were wortmannin, rapamycin and manumycin A. PD098059 was purchased from New England Biolabs (Berverly, MA). Polyclonal antibody (αY91), prepared in rabbits, against the synthetic peptide containing the triple tyrosine residues of Erk1 which recognize both Erk1 and Erk2 was provided by Dr. T.Kadowaki (University of Tokyo) [19]. Antibodies to phosphorylated Erk, phosphorylated (Thr421/Ser424) p70S6 kinase, and phosphorylated protein kinase B (PKB/Akt) were purchased from New England Biolabs. Antiphosphotyrosine antibody was obtained from Transduction Laboratories (Lexington, KY). [$^3$H]thymidine (74.0GBq/ml) and [$\gamma^{32}$P]ATP (222 TBq/mmol) were purchased from Dupont-New England Nuclear (Boston, MA).

### *Cell culture*

A10 cells were obtained from American type collection (ATCC) (Rockville, MD). This cell line was derived form the thoracic aorta of DBIX embryonic rats and possesses many of the characteristics of VSMC [20]. The cells were maintained in Dulbecco's modified Eagles medium (DMEM) supplemented with 10% fetal calf serum (Nakarai Chemical, Tokyo) in a humidified atmosphere of 95% air and 5% $C0_2$. All experiments were performed with cells from passages 5-20.

### *DNA synthesis*

The cells were grown to subconfluence in multiwell plastic plates. The medium was removed and replaced with serum-free DMEM supplemented with 0.1% bovine serum albumin (BSA). After 24h, the cells were exposed to the indicated concentrations of insulin with or without PDGF for 24h, in the presence of 1 μCi(7.4x$10^4$ Bq)/ml [$^3$H]thymidine (20Ci/mmol; New England Nuclear, Boston, MA). Cultures were washed twice with ice-cold phosphate-buffered saline (PBS) and precipitated with ice-cold 10% trichloroacetic acid (TCA). DNA synthesis in the cells (per $10^5$ cells) was determined by the extent of [$^3$H]thymidine incorporation into TCA-insoluble fractions.

### *Cell processing*

At the indicated period after stimulation, the medium was removed and the cells washed once with ice-cold PBS and scraped on ice into lysis buffer (25 mmol/l Tris/HCl, pH 7.4, 25 mmol/l NaCl, 1 mmol/l sodium orthovanadate, 10 mmol/l NaF, 0.5 mmol/l EGTA, 10 mmol/l sodium pyrophosphate, 10 nmol/l okadaic acid, 1 mmol/ PMSF, 1% Triton-X). The suspension was sonicated and the tubes were centrifuged at 15,000 x g for 60min at 4°C. The resultant supernatant was used for measurement of signaling components. The protein concentration was determined with a Bio-Rad Protein Assay kit (Bio-Rad Laboratories, Richmond, CA).

### *Kinase assay*

MAP kinase activity in cell extracts was measured by the ability to phosphorylate myelin basic protein (MBP). The procedure was essentially as previously described [19]. The cell extracts (200-400 μl) were incubated with αY91 in the presence of 0.1% SDS for 2h on ice, and 50 μl of a 50% (v/v) suspension of protein A-Sepharose were added and the incubation was continued for another for 3h at 4°C. The immunoprecipitated complex was washed twice with buffer A containing 25 mmol/l Tris-HCl (pH 7.4), 25 mmol/l NaCl, 1 mmol/l EGTA, and 1 mmol/l sodium orthovanadate,

10 mmol/l NaF, 10 mmol/l sodium pyrophosphate and 10 nmol/l okadaic acid. The immunoprecipitates were washed twice with buffer A, and resuspended in 50 µl of kinase buffer (25 mmol/l Tris-HCl , pH7.4, 10 mmol/l $MgCl_2$, 1 mmol/l dithiothreitol, 40 µmol/l ATP, 2 µCi of [$\gamma$-$^{32}$P]ATP, 2 µmol/l cAMP dependent-protein kinase inhibitory peptide, 0.5 mmol/l EDTA) containing 25 µg MBP (Sigma, St Louis, MO) and incubated at 25°C for 15min. The reaction was terminated by adding 15 µl of stopping solution containing 0.6 mol/l HCl, 1 mmol/l ATP and 1% BSA. Aliquots (15 µl) of the supernatants were spotted onto 1.5 x 1.5 cm squares of p81 (Whatman International, Maidstone, UK), washed 5 times for 5min each in 0.5% phosphoric acid, washed in acetone, dried and counted using Cerenkov counting. In some experiments, MAP kinase activity in selected samples was measured using myelin basic protein (MBP) containing polyacrylamide gels (in gel assay) as previously described [21]. Twenty µl of the cell extract (protein 20µg) was electrophoresed on SDS-polyacryalmide gel containing 500 mg/l MBP. After the enzymes in the gel had been denatured and then renatured, the gel was incubated with 10 ml of 40 mmol/l of Hepes, pH 8.0, containing 0.5 mmol/l EGTA, 10 mmol/l $MgCl_2$, 2 µmol/l protein kinase inhibitor, 40 µmol/l ATP, and 25 µCi of [$\gamma$-$^{32}$P]ATP at 25 C for 1h. The gel was washed with a 5% (w/v) trichloroacetic acid (TCA) solution containing 1% sodium pyrophosphate, and phosphorylated MBP was identified by autoradiography of the dried gel. Raf-1 kinase activity was determined using gluthathione transferase (GST)-MEK as a substrate [22]. GST-MEK had been bacterially produced and purified on glutathione-agarose beads. Forty µl of cell extracts were incubated for 30min at 25°C in buffer B (25 mmol/l Tris-HCl, pH 7.4, 10 mmol/l $MgCl_2$, 30 mmol/l DTT, 1mmol/l MnCl2, 1mmol/l EGTA) containing 5 µCi [$\gamma$-$^{32}$P-ATP] and GST-MEK (10 µg), and the mixture was incubated for 30min at 25°C. Then, 500 µl of a glutathione-Sepharose 4B bead suspension (Pharmacia Biotech, Uppsala, Sweden) in buffer A were added to collect GST-MEK. After 30min on ice, the Sepharose beads were washed once in buffer B and then boiled for 3min in Laemmli sample buffer, rapidly sedimented, and the supernatant was then used for 8% SDS-PAGE. Following electrophoresis, phosphorylated GST-MEK was identified by autoradiography. PI3K activity was measured on anti-phosphotyrosine immune complexes, essentially as described previously [23]. The reactions were initiated by the addition of 50 nmol/l ATP, 5 mmol/l $MgCl_2$ and 2 µCi of [$\gamma$-$^{32}$P] ATP and stopped after 20 min at 30C with 10µl of 4 mol/l HCl and 250µl of chloroform/methanol (1:1, v/v). The lower organic phase was washed with 150 µl of methanol/1M HCl (1:1, v/v) and dried. Phospholipids were resuspended in 10 ml of chloroform, separated by TLC on aluminum-backed silica gel 60 plates pretreated with 1% potassium oxaloate in the solvent system chroroform/methanol/acetone/acetic acid water (60:20:23:18:11; vol/vol), and the detection and quantification of [$^{32}$P]-PI3P were accomplished by autoradiography.

### *Western blot*

The cell extract (20 µg protein) in Laemmli sample buffer (62 mmol/l Tris-HCl buffer, pH 6.8, in the presence of 2.0% sodium dodecyl sulfate, 10% glycerol, and 0.05% bromophenol blue) was heated for 5min at 95°C, and resolved on 10% SDS-gel in the presence of a reducing agent. The proteins were then transferred onto a nitrocellulose sheet (Japan Bio-Rad, Tokyo) in transfer buffer (25 mmol/l Tris-HCl/192 mmol/l glycine/20% methanol) with Trans-Blot apparatus (Bio-Rad). Protein binding sites were blocked by incubating the sheets for 1h with 5% skim milk powder in TTBS buffer (20 mmol/l Tris-HCl, pH 7.5 /137

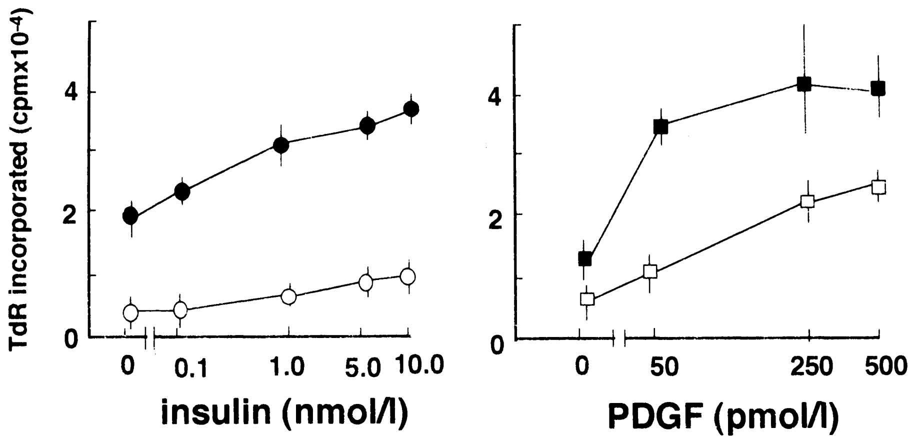

FIGURE 1
Synergism between insulin and PDGF in stimulating DNA synthesis.
*Left:* Subconfluent A10 cells in serum-free medium were treated with the indicated concentrations of insulin without (○) or with 250 pmol/l PDGF (●).
*Right:* The cells were treated with PDGF alone (□) or in the presence of 1 nmol/l insulin (■). After 16 h of incubation, [$^3$H]-thymidine incorporated into DNA was measured as described in the text. The values are means±SD of triplicate determinations from one representative experiment out of three.

mmol/l NaCl containing 0.1% Tween 20). The sheets were washed three times and then incubated overnight with antibodies to MAPK (Erk), phosphospecific MEK, p70S6 Kinase (P70S6K), or protein kinase B (PKB/Akt). The immune-complex was visualized with an enhanced chemiluminescence (ECL) system (Amersham, Buckinghamshire, UK) according to the manufacturer's manual using Kodak XAR film. The bands on the film were quanfied densitometrically.

### *Adenovirus-mediated gene transfer*

The cDNA plasmid for dominant negative Ras (Asn-17) was provided by Dr. T. Kadowaki T (University of Tokyo). The recombinant adenoviruses AdexCALacZ and AdexCAdnRas were constructed by homologous recombination between the expression cosmid cassette and the parental viral genome, and adenovirus-mediated gene transfer was carried out as previously described [24]. When the adenovirus AdexCALacZ was applied at 3x10$^7$ PFU per cm$^2$ dish, LacZ was expressed in more than 90% of the cells 24h after transfection. We applied this dose and control cells were transfected with AdexCALacZ virus. An adenovirus vector encoding a dominant negative mutant Akt (AxCAAkt-AA) in which the phosphorylation sites (Thr308 and Ser473) were replaced by alanine was kindly provided by Drs. M. Kasuga and W. Ogawa (Kobe Univerisity). A10 cells were infected with the adenovirus vectors as described [25]. The cells were used 24-48 h after the transfection.

### *Statistical analysis*

The differences between groups were determined by ANOVA (Fisher's multiple comparisons test) using the STATView program (Abacus, Berkeley, CA). Differences were considered significant when the p-value was less than 0.05. The values presented are mean±SD unless otherwise stated.

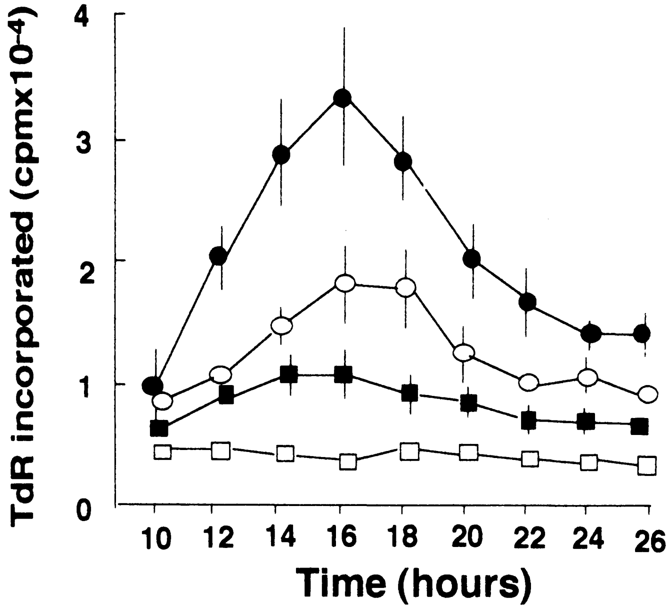

FIGURE 2
Time course of DNA synthesis stimulated by PDGF in the presence or absence of insulin.
A10 cells were incubated with 50 pmol/l of PDGF alone (○), PDGF plus 1 nmol/l of insulin (●), insulin alone (■) or medium alone (□) for the indicated periods. [$^3$H]-thymidine was added during the last 2 h. The values are means±SD of triplicate determinations.

### RESULTS

### *Synergism of insulin and PDGF in mitogenesis*

Insulin showed poor mitogenic activity in A10 cells, and DNA synthesis was detected only with insulin concentrations higher than 1.0 nmol/l. However, in the presence of 250 pmol/l PDGF, low concentrations of insulin (0.1-1.0 nmol/l) dose-depedendently stimulated DNA synthesis (Fig.1 left). Likewise, the presence of insulin (1.0 nmol/l) significantly potentiated PDGF-stimulated DNA (Fig.1 right); DNA synthesis induced by 50 pmol/l PDGF increased by more than 3-fold in the presence of insulin.

The apparent synergism between insulin and PDGF was not due to the difference in the time course of DNA synthesis between cells stimulated by PDGF alone versus those exposed to PDGF plus insulin. In both cases, peak [$^3$H]-thymidine incorporation was obtained at 16-18 h after stimulation (Fig. 2).

### *Signaling by PDGF*

We first studied the signaling pathways elicited by PDGF and their roles in DNA synthesis in A10 cells. As shown in Fig.3, PDGF phosphorylated and activated MAP kinases (Erk1 and Erk2) within 5min and the peak activity was generally seen at 10-20 min after stimulation. The activity then decreased, followed by a slight increase at 3-6 h. Generally, the activity remained above basal levels for more than 12h, which is similar to the result reported for human VSMC [26]. As reported for other types of cells, PDGF also stimulated Akt and p70S6K phosphorylation in A10 cells?

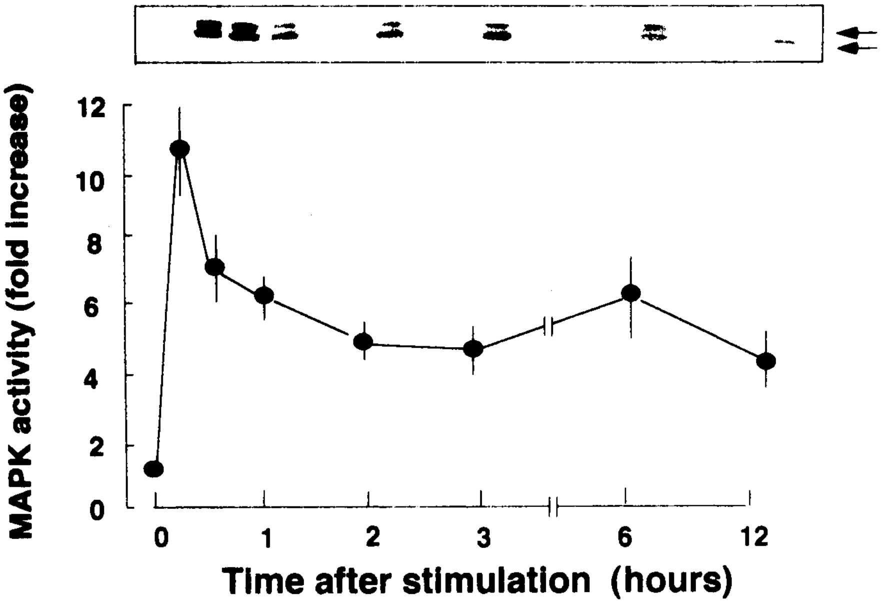

FIGURE 3
Effect of PDGF on MAP kinase activation and phosphorylation.
The cells cultured in serum-free medium for 24 h were challenged with 50 pmol/l PDGF for the indicated periods. MAP kinase activity was determined by phosphorylation of MBP as described in the text (mean±SD of triplicate determinations). Western blots results using antibodies to phosphorylated Erks are shown at the top (representative of four experiments). Arrows indicate phosphorylated Erk 1 (44KDa) and phosphorylated Erk 2 (42KDa).

(Fig. 4). Phosphorylation of both kinases was evident within 10min. In most studies, the extent of Akt phosphorylation returned to basal levels by 12 h, whereas that of p70S6K remained above basal levels for longer periods.

It has been shown that activations of both Akt and p70S6K is, at least in part, PI3K-dependent [16, 26]. Pretreatment of cells with wortmannin (100 nmol/l), a PI3K inhibitor, resulted in a significant decrease (32±6 % of control cells; in 3 experiments) in PDGF-stimulated PI3K activity (Fig. 5). Likewise, phosphorylations of Akt and p70S6K were significantly attenuated by wortmannin.

In cells transfected with dnRas, PDGF-stimulated PI3K activation decreased slightly, but significantly ($p<0.05$), to 74±3 % of that in control cells transfected with the vector alone (LacZ) in 3 experiments. Phosphorylations of Akt and p70S6K also tended to be decreased as compared to those in control cells, but the differences did not reach statistical significance.

To ascertain the roles of the MAPK cascade and PI3K-mediated signaling pathways in the mitogenic activity of PDGF, the effects of several inhibitors of the MAPK cascade or PI3K-mediated pathways on the PDGF-stimulated DNA synthesis were studied, and the results were correlated with the stimulated MAPK activities (Fig. 6). Treatment of A10 cells with PD98059 (30 μmol/l), a MEK inhibitor, almost completely abolished PDGF-stimulated MAPK activity, while decreasing DNA synthesis by only 40±4 %. The potentiating effects of insulin on PDGF-induced DNA synthesis and MAPK activation were not seen in cells treated with PD98059 (data not shown).

In cells transfected with dominant negative Ras (dnRas), both DNA synthesis and MAPK activation by PDGF, in the presence or absence

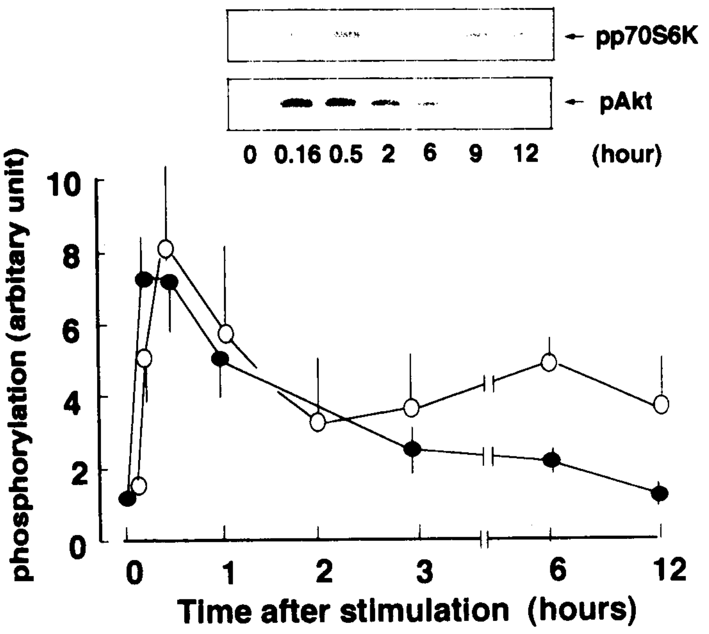

FIGURE 4
Effects of PDGF on phosphorylations of Akt and p70S6K
The cells were treated with PDGF (250 pmol/l) for 10min, and cell lysates were subjected to Western blot using antibodies against phosphorylated Akt or 70S6K. Representative Western blots are shown at the top. Results of densitometric analysis of Western blots in 4 experiments (open circle: pp70S6K, closed cicle: pAkt ) are shown in the botom panel (means±SD).

of insulin were almost completely abolished, while the responses in cells transfected with the vector alone were comparable to those in control cells. Similarly, treatment with manumycin A, (10 mmol/l for 8h) an inhibitor of transferase, significantly inhibited both DNA synthesis and MAPK activation, and the responses to PDGF were not different from those to PDGF plus insulin (data not shown).

Rapamycin (30 nmol/l), reportedly a specific inhibitor of p70S6K, also potently inhibited PDGF-stimulated mitogenic activity, but showed no effects on PDGF-stimulated MAPK activity. Treatment with wortmannin (100 nmol/l) significantly ($p<0.01$) inhibited MAPK activity (70±4 % of control) and DNA synthesis (56±3%). Transfection of dominant negative Akt (dnAkt) resulted in 30-50% decreases in both basal PDGF-stimulated DNA synthesis and MAPK activation, but the percent increases (stimulated/basal) were not different from those in cells transfected with the vector alone (data not shown). Cells transfected with the mutant Akt, however, easily detached from the bottoms of dishes and died, most likely via apoptosis.

### *Effect of insulin on PDGF-induced signaling*

We next studied whether insulin modifies the signaling pathways stimulated by PDGF. The effects of insulin, PDGF and insulin plus PDGF on activation of Raf-1, which functions upstream from MEK1 (MAPK kinase), and on the downstream MAPK were evaluated (Fig. 7). Although insulin at 1 nmol/l alone activated neither Raf-1, nor MAPK, it significantly ($p<0.05$) potentiated and sustained the activitions of the kinases stimulated by PDGF (50 pmol/l). In contrast, there was no synergism

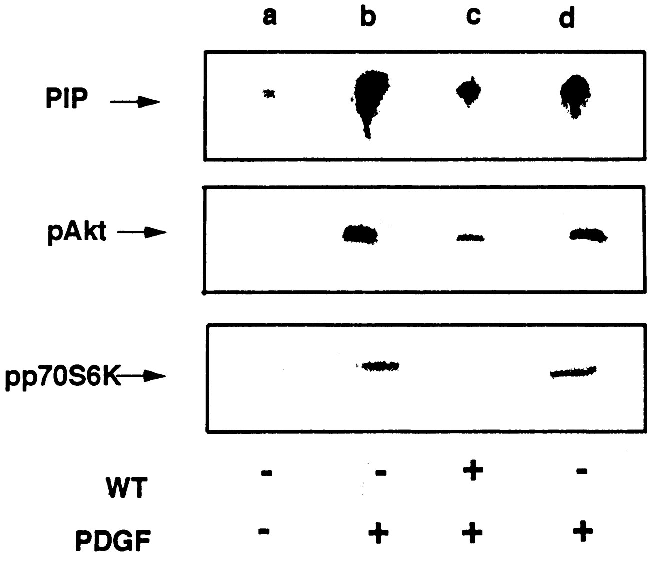

FIGURE 5
Wortmannin-dependent phosphorylation of Akt and p70S6K
The cells were pretreated with (lane c) or without (lanes a, b) 100 nmol/l of wortmannin (WT). They were then stimulated with 250 pmol/l of PDGF for 15min. Cells transfected with dnRas were similarly stimulated with PDGF (lane d). The cell lysate was immunopecipitated with antiphosphotyrosine antibody and PISK was determined as described in the text. A representative autoradiograph, from one of 4 experiments, is shown in the top panel. In parallel experiments, phosphorylations of Akt and p70S6K were quantified by Western blotting (middle and bottom panels).

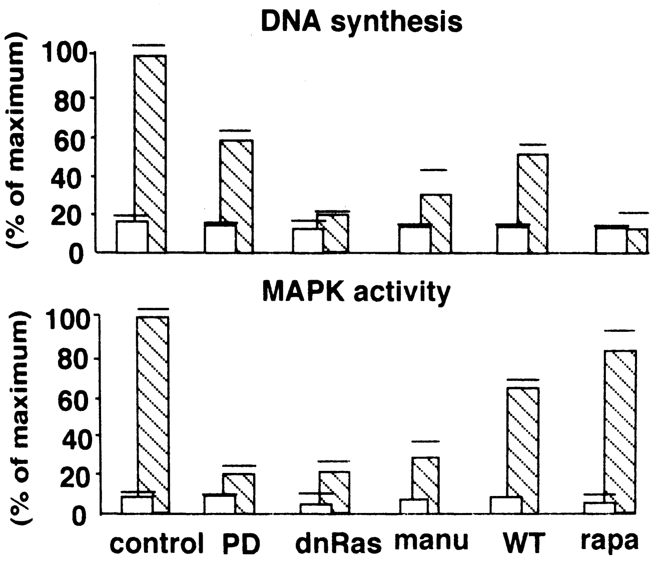

FIGURE 6
Relation between PDGF-stimulated MAPK activity and DNA synthesis.
The cells were pretreated with or without (control) PD98059 (PD, 30 µM), wortmannin (WT, 100 nmol/l), or rapamycin (rapa, 30 nmol/l) for 30min ,or with manumycin (manu, 10 µmol/l) for 8h, before stimulation with 250 pmol/l PDGF. The cells transfected with dnRas were also tested for the ability to respond to PDGF. DNA synthesis was measured as in Fig. 1. MAPK activity was measured by immune complex kinase assay in cells stimulated with PDGF for 10min. The results shown are the mean±SD (horizontal bar) of four experiments. Open and hatched columns represent the basal and post-PDGF stimulated values, respectively. The values are significantly (a, p<0.01; b, p<0.05) lower than those in non-pretreated control cells.

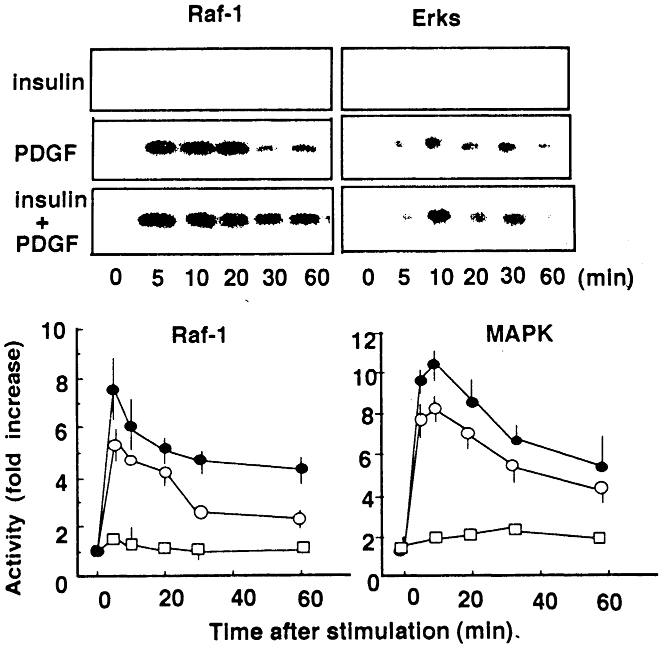

FIGURE 7
Effect of insulin on PDGF-induced MAPK activation
A10 cells were stimulated with 1nmol/l insulin alone (❑), 250 pmol/l/ PDGF alone (○) or insulin plus PDGF (●) for the indicated periods. Raf-1 activity was measured as the extent of phosphorylation of GST-MEK. MAPK activity was determined by kinase assay using MBP as a substrate. Data are expressed as means±SD of triplicate determinations. Activities of both MAPK and Raf-1 stimulated by PDGF plus insulin were significantly ($p<0.05$) higher than those stimulated by PDGF alone. Autoradiographs of GST-MEK (left) and MBP (right) in gel (in gel assay; as described in Materials and Methods).

between insulin and PDGF in the abilities to stimulate PI3K-dependent pathways (Fig. 8); low concentrations of insulin only minimally stimulated phosphorylations of PKB/Akt and p70S6K, and did not influence the PDGF-stimulated phosphorylations at any time point. In selected samples, kinase activities of p70S6K stimulated PDGF alone and those of PDGF plus insulin were measured using synthetic S6 peptide as a substrate [27]. PDGF alone stimulated activation of p70S6K by 5.6±0.3 fold at 10min, and by 5.5±0.2 fold at 30min, respectively in three experiments. The values were comparable to those in cells stimulated by PDGF in the presence of 1.0 nmol/l insulin (4.6±1.5 fold at 10 min, and 4.4±0.5 fold at 30 min).

### DISCUSSION

The data presented herein show that physiological concentrations of insulin (0.1-1 nmol/) have little mitogenic activity alone while potentiating PDGF-induced DNA synthesis in A10 cells. Our goal was to determine how insulin potentiates the mitogenic effect of PDGF. To address this question, we first studied which signaling pathways are involved in DNA synthesis stimulated by PDGF. Consistent with a previous report [28], PDGF-stimulated DNA synthesis and MAPK activation were significantly reduced by transfection with dnRas. Furthermore, manumycin A which inhibits farnesylaion of Ras, attenuated the responses. Signaling pathways mediated by Ras are quite

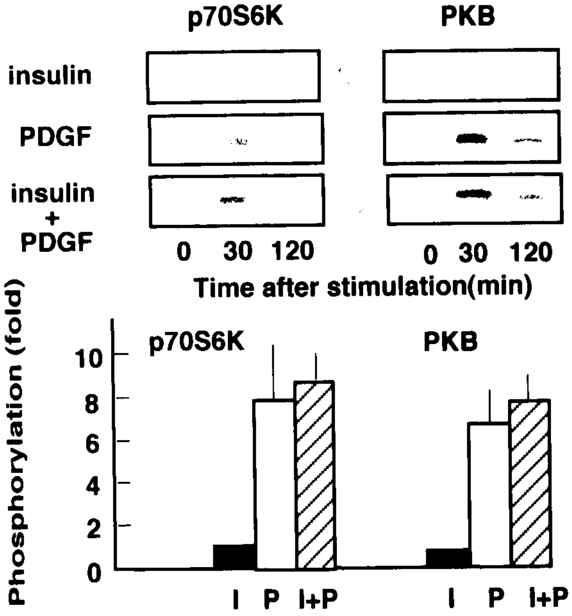

FIGURE 8
Effects of insulin on PDGF-induced Akt and p70S6K phosphorylations.
Upper panel: The cells were treated as in Fig.7. Phosphorylated PKB/Akt (right) and p70S6K (left) were determined by Western blotting as in Fig. 4. Western blots shown at the top are representative of 4 experiments.
Lower panel: Fold increases (stimulated/basal) in phosphorylations of p70S6K (left) and PKB/Akt (right) in the cells stimulated with PDGF (P: 250 pmol/l), insulin (I:1 nmol/l) or PDGF plus insulin (P+I) for 20min were quantified densitometryically and are shown at the bottom (the means±SD of three experiments). There were no significant differences in phosphorylations of either p70S6K or Akt in the cells stimulated with PDGF alone versus those stimulated with PDGF plus insulin for 6 h (data not shown).

diverse [29], but loss of mitogenic responses to PDGF in cells transfected with dnRas may be accounted for, at least in part, by failure of the cells to activate the MAPK cascade. Supporting this, treatment of A10 cells with PD98059, an inhibitor of MEK significantly attenuated PDGF-induced DNA synthesis. Furthermore, Robinson et al have reported that treatment of rat aortic smooth muscle cells with antisense oligonucleotides directed against MAPK almost completely blocked PDGF-stimulated DNA synthesis [30]. However, the inhibition of DNA synthesis by PD98059 in A10 cells was not complete at the concentration that completely abolished PDGF-induced MAPK activation in these cells. Thus, MAPK activation is required but not sufficient for full mitogenic activity of PDGF.

We also showed PDGF-stimulated MAPK activation to be partly inhibited by wortmannin, an inhibitor of PI3K. Reports on the effect of wortmannin on MAPK activation are inconsistent, depending on cell type, or ligand [31]. The mechanism of MAPK inhibition by wortmannin is not clear at present, but grb2 or PKC family members downstream from PLCγ may be involved [31, 32].

PDGF also stimulated phosphorylations of p70S6K and Akt in a wortmannin-sensitive manner, as reported for several cell types. The potent inhibition of DNA synthesis by rapamycin suggests that p70S6K plays an important role in mitogenesis during progression through the G1 phase of the cell cycle [33].

The activation of p70S6K by PI3K is indirect, and nonclassical protein kinase C (nPKC) or Akt activated by PI3K, may be involved [18]. Furthermore, classical protein kinase C (cPKC), produced by phospholipase C-γ (PLC-γ) or TPA, is able to activate p70S6K [27, 34]. The role of Akt in the mitogenic response to PDGF appars to be minor, because transfection with dominant negative Akt did not have significant effects on DNA synthesis although the transfection did accelerate cell death. Taken together, these results indicate that both the MAPK cascade and p70S6K are involved in PDGF-stimulated DNA synthesis in A10 cells.

We next studied how insulin affects these signaling pathways and thereby enhances DNA synthesis induced by PDGF. Low concentrations of insulin enhanced PDGF-stimulated MAPK, but not Akt or p70S6K activation. The results suggest that the potentiating effects of insulin on DNA synthesis are largely mediated via MAPK pathway. This is supported by the observation that the synergistic effect of insulin was not seen when the cells were pretreated with PD98059 or manumycin. In separate experiments, insulin also enhanced both EGF-stimulated DNA synthesis and MAPK activation (data not shown), and the potentiating effect of insulin was not specific to PDGF.

The enhancing effect of insulin was also seen in PDGF-stimulated Raf-1 activation. Raf-1 is a serine/threonine kinase which functions upstream from MAPKK (MEK) and is activated by interacting with activated Ras (Ras-GTP), and farnesylation of Ras is required to for its translocation to the plasma membrane and subsequent activation [34]. Insulin has been shown to increase the amount of farnesylated Ras via promoting activation of farnesyltransferase, and thereby augments the pool of cellular Ras available for activation by growth factors [11, 35]. Since Ras is a key upstream factor controlling Raf-1 activity, ,increased availability of Ras may potentiate PDGF-stimulated sequential activation of Raf-1, MEK and MAPK and thererby DNA synthesis. The lack of an insulin effect on PDGF-stimulated Akt or p70S6K could be explained by activation of these kinases without requiring Ras; transfection of dnRas had a minimal effect on PDGF-stimulated activation of these kinases.

In conclusion, insulin, at pathophysiologically relevant concentrations, potentiates PDGF-stimulated activation of the mitogenic response in A10 cells. The potentiating effect of insulin occurs, at least in part, via enhanced activation of the MAPK cascade. These results support the view that endogenous hyperinsulinemia may accelerate proliferation of VSMC and thereby the development of atherosclerosis. Inhibitors of MAPK activation are potential therapeutic agents which may retard the progression of atherosclerosis in hyperinsulinemic patients.

### *Acknowledgments*

The present work was supported by the grants from the Ministry of Health and Welfare, and the Ministry of Education (Tokyo, Japan). The authors thank Drs. T.Kadowaki, M. Kasuga and W.Ogawa for their help. The support by the Study Group of Pediatric and Adolescent Diabetes Japan is also greatly appreciated.

### REFERENCES

1. Ross, R., Glomset, J., Kariya, B. and Harker, L. (1974). A platelet-dependent serum factor that stimulates the proliferation of arterial smooth muscle cells in vitro. *Proc. Natl. Acad. Sci. USA*, **71**, 1207-1210.
2. Jawien, A., Bowen-Pope, D.F., Lindner, V., Schwartz, S.M. and Clowes, A.W. (1992). Platelet-derived growth factor promotes smooth muscle migration and intimal growth thickening in a rat model of balloon angioplasty. *J. Clin. Invest.*, **89**, 507-511.
3. Bornfeldt, K.E., Arnqvist, H.J. and Capron L. (1992). In vivo proliferation of rat vascular smooth muscle in relation to diabetes mellitus insulin-like growth factor and insulin. *Diabetologia*, **35**, 104-108.

4. Grainger, D.J., Witchell, C.M., Weissberg, P.L. and Metcalfe, J.C. (1994). Mitogens for adult rat aortic vascular smooth muscle cells in serum-free primary culture. *Cardiovasc. Res.*, **28**, 1238-1242.

5. King, G.L., Goodman, D., Buzney, S., Moses, A. and Kahn, C.R. (1985). Receptors and growth promoting effects and insulin-like growth factors on cells from bovine retinal capillaries and aorta. *J. Clin. Invest.*, **75**, 1028-1036.

6. Bornfeldt, K.E., Raines, E.W. and Nakano, T. (1994). Insulin-like growth factor-I and platelet-derived growth factor-BB induce directed migration of human arterial smooth muscle cells via signalling pathways that are distinct from those of proliferation. *J. Clin. Invest.*, **93**, 1266-1274.

7. Bilato, C., Pauly, R.R. and Medillo, G. (1995). Intracellular signalling required for rat vascular smooth muscle cell migration. Interaction between basic fibroblast growth factor and platelet-derived growth factor. *J. Clin. Invest.*, **96**, 1905-1915.

8. Emoto, N., Onose, H., Yamada, H., Minami, S., Tsushima, T. and Wakabayashi, I. (1998). Growth factors increase pericellular proteoglycans independently of their mitogenic effects on A10 rat vascular smooth muscle cells. Int. J. Biochem. and Cell, 30, 47-54. [9] Stolar, M.W. (1988) Atherosclerosis in diabetes: the role of hyperinsulinemia. *Metabolism*, **37**( 2 Suppl 1), 1-9.

10. Stout, R.W. (1990). Insulin and atheroma, 20-yr perspective. *Diabetes Care*, **13**, 631-654.

11. Goalstone, M.L., Natarajan, R., Standley, P.R, Walsch, M.F., Leitner, J.W., Carel, K., Scott,S., Nadler, J., Sowers, J.R. and Draznin, B. (1998). Insulin potentiates platelet-derived growth factor action in vascular smooth muscle cells. *Endocrinology*, **139**, 4067-4072.

12. James, D.E. (2000). Signalling through the insulin receptor. *Curr. Op. Cell. Biol.*, **12**, 222-228.

13. Clesson, R. and Welsh, L. (1994). Platelet-derived growth factor receptor signals. *J. Biol. Chem.*, **269**, 32023-32026.

14. Seger, R.and Krebs, A.G. (1995). The MAPK signaling cascade. *FASEB J.*, **9**, 726-735.

15. Widmann, C., Gibbson, S., Jappe, M.B. and Johnson G.L. (1999). Mitogen-activated protein kinase: Conservation of a three-kinase module from yeast to human. *Physiol. Rev.*, **79**, 143-180.

16. Leevers S.J, Vanhaesebroeck, B, Waterfield, M.A (1999). Signaling through phosphoinositide 3-kinases: the lipids take centre stage. *Curr. Op. Cell Biol.*, **11**, 219-225.

17. Downward, J. (1998). Mechanism and consequences of activation of protein kinase B/Akt. *Curr. Opin. Cell. Biol.*, **10**, 262-267.

18. Chou, M.M. and Blenis, J. (1995). The 70kDa S6 kinase: regulation of a kinase with multiple roles in mitogenic signalling. *Curr. Opin. Cell Biol.*, **7**, 806-814.

19. Tobe, K., Kadowki T, Hata, K., Gotoh, Y., Kosako, H., Matsuda, S., Tamemoto, H., Ueki, K., Akanuma Y., Nishida E., and Yazaki, Y. (1992). Sequential activation of MAP kinase acivator, MAP kinases, and S6 peptide kinase in intact rat liver following insulin injecion. *J. Biol Chem.*, **267**, 21089-21097.

20. Rao, R.S., Miano, J.M., Olson, E.N.and Seidel, C.L. (1997). The A10 cell line: a model for neonatal, neointimal, or differentiated vascular smooth muscle cells? *Cardiovasc. Res.*, **36**, 118-126.

21. Tobe, K., Kadowaki, T., Tamemoto, H., Ueki, K., Hara, K., Koshio, O., Momomura, K., Gotoh, Y., Nishida, E., Akanuma, Y., Yazaki, Y. and Kasuga, M. (1991). Insulin and 12-O-tetradecanoyl-phorbol-13-acetate activation of two immunologically distinct myelin basic protein/microtubule-associated protein 2 (MBP/MAP2) kinases via de novo phosphorylation of threonine and tyrosine residues. *J. Biol. Chem.*, **266**, 24793-24803.

22. Seko, Y., Tobe, K., Ueki, K., Kadowaki, T. and Yazaki, Y. (1996). Hypoxia and hypoxia/reoxygenation activate Raf-1, mitogen activated protein kinase kinases, and S6 kinase in cultured rat cardiac myocytes. *Circ. Res.*, **78**, 82-90.

23. Yamauchi, T., Tobe, K., Tamemoto, H., Ueki, K., Kaburagi,Y., Yamamoyo-Honda R., Takahashi Y., Yohizawa, F., Aizawa, S., Akanuma, Y., Sonenberg, N., Yazali. Y. and Kaodowaki, T. (1996). Insulin signaling and insulin actions in the muscles and livers of insulin-resistant, insulin receptor substrate 1-deficient mice. *Mol Cell. Biol.*, **16**, 3074-3084.

24. Miyake, S., Makimura, M., Kanegae, Y., Harasa, S., Sato, Y., Takamoti, K., Tokuda, C. and Saito, I. (1996). Efficient generation of recombinant adenovirus DNA-terminal protein complex and a cosmid bearing the full-length virus genome. *Proc. Natl. Acad. Sci. USA*, **93**, 1320-1324.

25. Kitamura, T., Ogawa, W., Sakaue, H., et al. (1998). Requirement for activation of the serine-threonine kinase Akt (protein kinase B) in insulin stimulation of protein synthesis but not of glucose transport. *Mol. Cell. Biol.*, **18**, 3708-3717.

26. Mii, S., Khalil, R.A., Morgan, K.G., Ware, J.A. and Kent, K.C. (1996). Mitogen activated protein kinase and proliferation of human vascular smooth muscle cells. *Am. J. Physiol.*, **270**, H142-H150.

27. Chung, J., Grammer, T.G., Lemon, K.P., Kazlaukas, A. and Blenis, J. (1994) PDGF- and insulin-dependent pp70S6K activation mediated by phosphatidylinositol 3-OH kinase. *Nature*, **370**, 71-75.

28. Yamazaki, T., Tobe, K., Hoh, E., Maemura, K., Kaida, T., Komuro, I., Tamemoto, H., and Kadowaki, T. (1993). Mechanical loading activates mitogen- activated protein kinase and S6 peptide kinase in cultured rat cardiac myocytes. *J. Biol. Chem.*, **268**, 12069-12076.

29. Irani, K., Herzlinger, S. and Finkel T. (1994). Ras proteins regulate multiple mitogenic pathways in A 10 vascular smooth muscle cells. *Biochem. Biophys. Res. Commun.*, **202**, 1252-1258.

30. Katz, M.E. and McCormick, F. (1997). Signal transduction from multiple Ras effectors. *Curr. Opin. Genet. Dev.*, **7**, 75-79.

31. Robinson, C.J., Scott, P.H., Allan, A.B., Jess, T., Gould, G.W. and Plevin R. (1996). Treatment of vascular smooth muscle cells with antisense phosphorothiolate oligodeoxynucleotides directed against p42 and p44 mitogen-activated protein kinases abolishes DNA synthesis in response to platelet derived growth factor. *Biochem. J.*, **15**, 320, 123-127.

32. Duckworth, B.C. and Cantley, L.C. ( 1997). Conditional inhibition of the mitogen-activated protein kinase cascade by wortmannin. *J. Biol. Chem.*, **272**, 27665-27670.

33. Conway, A-M., Rakhit, S., Pyne, S. and Pyne, N.J. (1999). Platelet-derived growth factor stimulation of the p42/p44 mitogen-activated protein kinase pathway in airway smooth muscle: role of pertussis-toxin-sensitive G-proteins, c-Src tyrosine kinases and phosphoinositide 3-kinase. *Biochem. J.*, **337**, 171-177.

34. Lane, H.A., Fernandez, A., Lamb, N.J.C. and Thomas, G. (1993) p70S6K function is essential for G1 progression. *Nature*, **363**, 170-172.

35. Kikuchi, A and Williams, L.T. (1994) The post translational modification of ras p21 is important for Raf-1 function. *J. Biol. Chem.*, **269**, 20054-20059.

36. Goalstone L, ML, Leithner JW, Wall K, Dolgonos L, Rother KI, Accili D. and Draznin, B (1998) Effect of insulin on farnesyl transferase. Specifity of insulin action and potentiation of nuclear effects of insulin-like growth factor-1, eipdermal growth factor, and plaetelet-derive growth factor. *J. Biol Chem.*, **273**, 23892-23896.

